# Good continuum of HIV care in Belgium despite weaknesses in retention and linkage to care among migrants

**DOI:** 10.1186/s12879-015-1230-3

**Published:** 2015-11-03

**Authors:** D. Van Beckhoven, E. Florence, J. Ruelle, J. Deblonde, C. Verhofstede, S. Callens, E. Vancutsem, P. Lacor, R. Demeester, J.-C. Goffard, A. Sasse, For the BREACH (Belgian Research on AIDS and HIV Consortium)

**Affiliations:** Epidemiology of Infectious Diseases Unit, Scientific Institute of Public Health, Rue J. Wytsman 14, 1050 Brussels, Belgium; Department of Clinical Sciences, Instituut Tropische Geneeskunde, Antwerp, Belgium; Institute of Experimental and Clinical Research (IREC), Unit of Medical Microbiology (MBLG), Université Catholique de Louvain, Brussels, Belgium; AIDS Reference Laboratory, Department of Clinical Chemistry, Microbiology and Immunology, Ghent University, Ghent, Belgium; Department of Internal Medicine, Universitair Ziekenhuis Gent, Ghent, Belgium; Department of Microbiology and Infection Control, Universitair Ziekenhuis Brussel, Brussels, Belgium; Department of Internal Medicine, Universitair Ziekenhuis Brussel, Brussels, Belgium; Department of Internal Medicine and Infectious Diseases, CHU de Charleroi, Charleroi, Belgium; Service of Internal Medicine, Hôpital Erasme, Brussels, Belgium

**Keywords:** HIV, Cascade, Continuum of care, Migrants, Belgium

## Abstract

**Background:**

The Belgian HIV epidemic is largely concentrated among men who have sex with men and Sub-Saharan Africans. We studied the continuum of HIV care of those diagnosed with HIV living in Belgium and its associated factors.

**Methods:**

Data on new HIV diagnoses 2007–2010 and HIV-infected patients in care in 2010–2011 were analysed. Proportions were estimated for each sequential stage of the continuum of HIV care and factors associated with attrition at each stage were studied.

**Results:**

Of all HIV diagnosed patients living in Belgium in 2011, an estimated 98.2 % were linked to HIV care, 90.8 % were retained in care, 83.3 % received antiretroviral therapy and 69.5 % had an undetectable viral load (<50 copies/ml). After adjustment for sex, age at diagnosis, nationality and mode of transmission, we found lower entry into care in non-Belgians and after preoperative HIV diagnoses; lower retention in non-Belgians and injecting drug users; higher retention in men who have sex with men and among those on ART. Younger patients had lower antiretroviral therapy uptake and less viral suppression; those with longer time from diagnosis had higher ART uptake and more viral suppression; Sub-Saharan Africans on ART had slightly less viral suppression.

**Conclusions:**

The continuum of HIV care in Belgium presents low attrition rates over all stages. The undiagnosed HIV-infected population, although not precisely estimated, but probably close to 20 % based on available survey and surveillance results, could be the weakest stage of the continuum of HIV care. Its identification is a priority along with improving the HIV care continuum of migrants.

**Electronic supplementary material:**

The online version of this article (doi:10.1186/s12879-015-1230-3) contains supplementary material, which is available to authorized users.

## Background

The use of ART (antiretroviral therapy) in HIV-infected patients has shown its efficiency not only in improving the individual outcomes of patients but also in reducing the transmission of HIV [1–3]. A persisting challenge of the HIV epidemic is reaching the highest proportion of overall viral suppression among people living with HIV (PLHIV) in order to impact HIV transmission [3, 4].

In Belgium the epidemic is largely concentrated among men who have sex with men (MSM) - mainly of Belgian or European nationality - and Sub-Saharan African (SSA) men and women. The number of annual new HIV diagnoses increased by half between 1997 and 2003 and has since remained stable at 1000 to 1200 new cases per year [5]. The number of PLHIV has steadily increased since introduction of ART. Recent surveys conducted in two large Belgian cities have shown an HIV prevalence among MSM ranging from 6 [6] to 12 % (C. Noestlinger, personnal communication, March 2015). In a survey among SSA migrants in Antwerp, HIV prevalences of 3 % in men and 6 % in women were found [7].

Services for HIV testing in Belgium are available either in primary care, secondary care (specialized outpatient clinics), hospitals and decentralized projects. Three Aids Reference Centers (ARC) are funded to perform low threshold and free anonymous testing. Outreach programs are set up to better target HIV high risk groups. These programs are organized by the ARCs or through a collaborative effort with NGOs working with the specific target population. Compared to other European countries [8], Belgium has a high and relatively stable rate of HIV testing, with a total of 695.433 HIV tests performed (62 per 1000 inhabitants) in 2013.

Access to HIV care in Belgium is, in principle, ensured to all HIV-infected individuals through the compulsory national health insurance. Antiretroviral therapy is reimbursed when a patient’s CD4 count is below or equal to 500 cells/mm^3^ or below 25 % and in case of clinical symptoms. There is no national guideline for HIV care, clinicians follow the guidelines of the European AIDS Clinical Society for clinical and laboratory monitoring [9]. Undocumented migrants may access care through a procedure called urgent medical help [10]. Nevertheless, the key populations in the Belgian HIV epidemic, MSM and migrants, differ in socio-demographic characteristics such as nationality, migrant status and social context which may influence their access to HIV testing and care.

In order to reach the best outcomes for HIV-infected individuals and potentially reduce HIV transmission, a continuum of HIV care services needs to be ensured. The HIV care cascade is an illustration of the continuum of HIV care built on estimates of the size of the HIV-infected population at different stages: infection, diagnosis, linkage to care, retention in care, ART uptake and viral load suppression. This approach allows pinpointing possible attrition along the continuum of care and may help to compare and prioritize prevention and care strategies [3]. These results also inform on the potential for domestic transmission of HIV through the estimation of the HIV-infected population without suppressed VL residing in the country. Several countries have already reported their domestic HIV care cascade [11–14]. These were built on available national surveillance data and survey results combined with estimates obtained by mathematical data modelling.

In this study, we estimated the proportion in each stage of the continuum of HIV care in Belgium among diagnosed PLHIV and analysed factors associated with attrition at each respective stage.

## Methods

### Data sources and setting

Estimates of the continuum of care from HIV diagnosis to undetectable viral load (VL) were calculated by analysing combined data from two surveillance systems managed by the Scientific Institute of Public Health (WIV-ISP): the registry of new HIV diagnoses and the Belgian HIV Cohort. The national registry of new HIV diagnoses [5] records all newly diagnosed confirmed HIV cases based on exhaustive reporting by the Belgian AIDS Reference Laboratories (ARLs). The Belgian HIV Cohort study [15] collects data on HIV-infected patients in care including data on viral load measurements recorded by the ARLs and CD4 counts and ART data recorded by the ARCs from 2007 onwards. All HIV-infected patients in medical care in Belgium have their VL analysed in the ARLs and around 75–80 % of patients have been followed in the ARCs in recent years. The WIV-ISP is the legal entity in charge of the HIV and AIDS surveillance activities (Royal decree of 8 October 1996). The HIV surveillance system was approved by the ethical committee of Ghent University hospital and authorized by the Privacy Commission. Strict attention to confidentiality is present at every stage of data collection, analysis and storage.

### Definitions

Proportions in each stage of the continuum of HIV care were estimated using the following definitions.

Linkage to HIV care was defined as having at least one VL or CD4 count recorded within 1 year of HIV diagnosis, with a window of 7 days for VL records to prevent incorrectly counting VL measurements performed at the time of diagnosis as initial HIV care visit.

Retention in HIV care was defined as the proportion of patients in care in 2010, those having one CD4 or VL measurement during that year, who had at least one record of CD4 or VL in 2011.

Proportions of patients on ART and with suppressed VL were measured among those in care in the ARCs in 2011. ART was defined as a record of ART prescription at the end of 2011. Suppressed VL was defined as the last measured VL <50 copies/mL.

### Continuum of HIV care

Proportions calculated for each stage of the continuum of HIV care were combined to obtain the distribution of the diagnosed HIV individuals living in Belgium in 2011 by stage of the continuum.

**Fig. 1:**
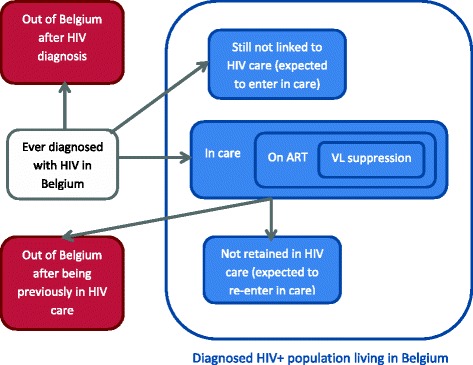
Diagram representing the distribution of the HIV diagnosed population within the continuum of HIV care

The total diagnosed HIV population living in Belgium was obtained by summing the estimated population retained in care in 2011 with the estimated number of HIV-positive persons diagnosed but not linked to care in 2011 and the estimated number of persons previously in HIV care but not retained in care in 2011. The distribution of the diagnosed HIV population through the stages of the continuum of care is schematized in Fig. 1.

Using 2007 data, we first estimated the proportion of patients that entered in care later than 1 year after diagnosis. We then applied this proportion of late entry into care to patients diagnosed in the years following 2007 to obtain an estimate of the total number of patients entering late in care. From this total we subtracted those who entered in care before the end of 2011 in order to obtain the estimated number of diagnosed patients not linked to care and expected to enter in care after 2011.

Next we estimated for the patients in care in 2007 and not retained in care after 1 year, the proportion who re-entered care later. Using a similar computation, the number of patients entering back in care was calculated for the years following 2007. From the cumulative total, we subtracted patients already re-entered in care by the end of 2011 in order to obtain the estimated number of patients not retained in care and expected to re-enter care after 2011.

Patients estimated to never enter or re-enter care were considered as having left the country. We also hypothesised that persons diagnosed before 2007 were not expected to enter in care after 2011.

The next stages of the continuum of HIV care were estimated by applying the proportions of patients on ART and with VL suppression to the population in HIV care in 2011.

### Data management and statistics

Databases were merged using a unique patient identifier. Identification of duplicate patient records was performed by comparing the following set of variables: gender, initials, date of birth, date of HIV diagnosis, presumed mode of transmission, nationality, place of residence and laboratory results.

Socio-demographic factors, time period since diagnosis and CD4 count at first medical consultation (±31 days) were analysed for association with each stage of the continuum of HIV care by multivariate logistic regression. Analyses were performed with Stata 10.1.

## Results

A total of 4117 individuals diagnosed with HIV between 2007 and 2010 were analysed for entry in care. Of 11,781 patients in care in 2010, 112 patients died before end of 2011, leaving 11,669 patients analysed for retention in care. ART uptake and VL levels were analysed for 9710 patients in care in the ARCs in 2011. Newly diagnosed individuals and patients in care shared the following similar socio-demographic characteristics (Table 1). Around two thirds were men and median age at diagnosis was approximately 35 years. Probable acquisition of HIV by sexual transmission was reported by more than 90 % of the patients with available information. Nearly half of the patients with reported nationality were Belgians and one third originated from countries in Sub-Saharan Africa.

**Table 1:** Baseline characteristics of HIV-infected individuals diagnosed between 2007 and 2010 and of patients in medical care in 2010 in Belgium

Characteristic	Individuals diagnosed with HIV, 2007–2010 (*N* = 4117)	Patients in medical care, 2010 (*N* = 11,669)
Sex		
Male	2715 (66.0 %)	7259 (62.2 %)
Female	1397 (33.9 %)	4409 (37.8 %)
Unknown	5 (0.1 %)	1 (0.0 %)
Age at diagnosis		
Median age (IQR), years	36 (29–44)	34 (28–41)
Way of transmission		
Heterosexual	1566 (38.0 %)	4560 (39.1 %)
MSM	1362 (33.1 %)	3149 (27.0 %)
IDU	63 (1.5 %)	217 (1.9 %)
Other	109 (2.7 %)	399 (3.4 %)
Unknown	1017 (24.7 %)	3344 (28.7 %)
Nationality		
Belgian	1401 (34.0 %)	4210 (36.1 %)
Sub-Saharan African	1107 (26.9 %)	3242 (27.8 %)
European	391 (9.5 %)	665 (5.7 %)
Other	259 (6.3 %)	548 (4.7 %)
Unknown	959 (23.3 %)	3004 (25.7 %)

**Fig. 2:**
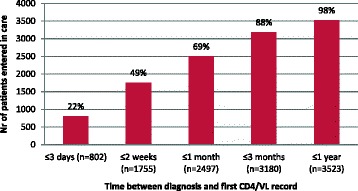
Distribution of delay between HIV diagnosis and first medical contact in HIV care (recorded CD4 or VL) among patients entered in care

Among newly diagnosed individuals, 87.0 % entered in HIV care within 1 year of diagnosis. This proportion increased over the years (2007: 84.5 %, 2008: 87.4 %, 2009: 87.0 %, 2010: 89.0 %; *p* < 0.001). Linkage to care was made within 3 months following HIV diagnosis for 88.1 % of those who entered in HIV care (Fig. 2). Median CD4 value at first medical contact was not significantly different between those who entered in care in the first 3 months following diagnosis (414 CD4 cells/mm^3^ (IQR: 248–595)) and those entered in care later (403 CD4 cells/mm^3^ (IQR: 221–585)).

Of those in care in 2010 and not reported to have died, 92.2 % were retained in care in 2011. Among those in care in the ARCs, 84.6 % were receiving ART, of whom 83.4 % were virally undetectable.

### Factors associated with entry, time to entry and retention in care

Univariate analyses show that entry and retention in care were lower among women and non-Belgians and higher among MSM (Table 2). Patients infected through injecting drug use (IDU) had lower retention in care. After adjustment for sex, age at diagnosis, nationality and mode of transmission, entry and retention in care remained lower among non-Belgians, retention was still higher among MSM and lower among IDU. Individuals tested for preoperative reasons had lower entry in care, they represented only 2.6 % of those with available data on reasons for testing, and presented significantly longer delay between diagnosis and entry in care than patients with all other reasons for testing (median time: 32 vs 14 days, *p* < 0.001). Linkage to care within 3 months of diagnosis was slightly lower among non-Belgians than Belgians (respectively 88.2 and 90.5 %; *p* = 0.049). Longer time period since HIV diagnosis and ART uptake were both independently associated with higher retention in care.

**Table 2:** Factors associated with entry in care (2007–2010) and retention in care (2010–2011) among HIV-infected patients in Belgium

	Entry in care			Retention in care		
Characteristic	Entry	OR for no entry (95 % CI)	AOR^a^ for no entry (95 % CI)	Retained	OR for no retention (95 % CI)	AOR^a^ for no retention (95 % CI)
Sex						
Male	88.8 %	1	1	92.9 %	1	1
Female	83.9 %	**1.52 (1.26–1.83)**	0.83 (0.58–1.19)	91.0 %	**1.30 (1.13–1.49)**	0.82 (0.66–1.04)
Age at diagnosis						
<40 years	87.1 %	1	1	94.1 %	1	1
≥40 years	87.0 %	1.01 (0.84–1.22)	0.91 (0.67–1.24)	93.9 %	1.02 (0.86–1.23)	1.01 (0.81–1.25)
Way of transmission						
Heterosexual	91.4 %	1	1	91.8 %	1	1
MSM	94.6 %	**0.61 (0.45–0.82)**	0.97 (0.62–1.51)	95.1 %	**0.56 (0.45–0.70)**	**0.59 (0.45–0.78)**
IDU	85.7 %	1.77 (0.85–3.66)	1.69 (0.71–4.05)	85.8 %	**1.87 (1.22–2.86)**	**2.01 (1.27–3.17)**
Nationality						
Belgian	95.7 %	1	1	95.8 %	1	1
SSA	88.8 %	**2.82 (2.05–3.88)**	**3.36 (2.14–5.27)**	92.8 %	**1.74 (1.42–2.12)**	**1.50 (1.16–1.93)**
European	88.5 %	**2.91 (1.94–4.35)**	**2.43 (1.52–3.90)**	91.7 %	**2.03 (1.48–2.78)**	**1.88 (1.35–2.61)**
Other	88.0 %	**3.04 (1.93–4.79)**	**3.01 (1.81–5.01)**	93.4 %	**1.58 (1.09–2.29)**	1.43 (0.97–2.10)
Reason for testing						
Patient’s request	93.3 %	1	1	95.0 %	1	1
Clinical arguments	93.5 %	0.97 (0.69–1.36)	0.90 (0.61–1.32)	94.3 %	1.15 (0.88–1.50)	1.01 (0.77–1.34)
Preoperative	79.2 %	**3.68 (2.01–6.73)**	**3.91 (2.03–7.53)**	93.1 %	1.42 (0.82–2.46)	1.31 (0.74–2.32)
Other	90.3 %	**1.51 (1.04–2.20)**	0.98 (0.63–1.53)	92.9 %	**1.46 (1.09–1.95)**	1.20 (0.89–1.63)
CD4 at first visit						
CD4≥350	/	/	/	93.2 %	1	1
CD4≥200 & <350	/	/	/	91.3 %	1.30 (0.90–1.86)	1.12 (0.74–1.69)
CD4 < 200	/	/	/	91.5 %	1.28 (0.87–1.86)	1.16 (0.76–1.77)
Time since HIV diagnosis						
<1 year	/	/	/	90.0 %	1	1
1 – <5 years	/	/	/	93.9 %	**0.59 (0.47–0.74)**	**0.55 (0.42–0.72)**
5 – <10 years	/	/	/	95.4 %	**0.44 (0.34–0.56)**	**0.42 (0.32–0.56)**
≥10 years	/	/	/	95.7 %	**0.41 (0.32–0.51)**	**0.40 (0.31–0.53)**
ART						
Not on ART end 2009	/	/	/	93.5 %	1	1
On ART end 2009	/	/	/	96.6 %	**0.50 (0.39–0.64)**	**0.45 (0.34–0.61)**

*p* < 0.05, statistically significant variables presented in boldface

^a^Adjusted for sex, age at diagnosis, nationality, way of transmission

### Factors associated with ART uptake and viral suppression among those on ART

Univariate analyses show that, younger age was associated with lower ART uptake and less viral suppression on ART, MSM had lower ART uptake and more viral suppression, those diagnosed for longer had higher ART uptake and higher VL suppression. Patients diagnosed for clinical reasons and with lower CD4 count at first contact had higher ART uptake. Sub-Saharan African patients on ART had less VL suppression (Table 3).

**Table 3:** Factors associated with ART uptake and VL suppression among HIV-infected patients in care in the ARCs (2011), Belgium

	ART uptake				VL suppression		
Characteristic	On ART	OR for no ART uptake (95 % CI)	AOR^a^ for no ART uptake (95 % CI)	AOR^b^ for no ART uptake (95 % CI)	Undetectable VL	OR for detectable VL (95 % CI)	AOR^a^ for detectable VL (95 % CI)
Sex							
Male	84.5 %	1	1	1	83.7 %	1	1
Female	84.9 %	0.97 (0.87–1.09)	**1.38 (1.14–1.67)**	1.16 (0.85–1.58)	82.9 %	1.06 (0.93–1.19)	0.84 (0.70–1.01)
Age at diagnosis							
<40 years	84.0 %	1	1	1	82.3 %	1	1
≥40 years	85.5 %	0.89 (0.78–1.01)	0.88 (0.76–1.02)	**0.74 (0.60–0.91)**	85.2 %	**0.81 (0.70–0.93)**	**0.83 (0.71–0.97)**
Way of transmission							
Heterosexual	85.9 %	1	1	1	82.6 %	1	1
MSM	81.8 %	**1.36 (1.19–1.55)**	**1.54 (1.27–1.88)**	0.98 (0.73–1.32)	84.8 %	**0.85 (0.74–0.98)**	0.88 (0.72–1.07)
IDU	85.6 %	1.02 (0.66–1.59)	1.01 (0.64–1.60)	0.75 (0.27–2.11)	82.5 %	1.01 (0.65–1.56)	1.04 (0.66–1.65)
Nationality							
Belgian	83.8 %	1	1	1	85.1 %	1	1
SSA	86.4 %	**0.81 (0.71–0.93)**	0.87 (0.73–1.05)	0.89 (0.65–1.21)	81.3 %	**1.31 (1.14–1.52)**	**1.25 (1.04–1.51)**
European	82.5 %	1.10 (0.88–1.37)	1.14 (0.91–1.44)	0.84 (0.61–1.15)	84.2 %	1.07 (0.83–1.38)	1.01 (0.77–1.32)
Other	81.9 %	1.14 (0.90–1.45)	1.13 (0.89–1.45)	1.17 (0.81–1.67)	81.1 %	1.33 (1.03–1.73)	1.21 (0.92–1.59)
Reason for testing							
Patient’s request	76.7 %	1	1	1	82.6 %	1	1
Clinical arguments	86.2 %	**0.53 (0.45–0.62)**	**0.57 (0.48–0.67)**	**0.77 (0.61–0.97)**	82.9 %	0.98 (0.82–1.18)	0.96 (0.79–1.16)
Preoperative	78.8 %	0.89 (0.62–1.27)	0.95 (0.64–1.40)	1.82 (0.95–3.52)	88.2 %	0.64 (0.38–1.07)	0.65 (0.38–1.11)
Other	83.5 %	**0.65 (0.54–0.78)**	**0.73 (0.60–0.89)**	0.92 (0.68–1.25)	83.7 %	0.93 (0.75–1.15)	0.87 (0.69–1.10)
CD4 at first visit							
CD4≥350	58.5 %	1	1	/	76.5 %	1	1
CD4≥200 & <350	88.0 %	**0.19 (0.15–0.25)**	**0.19 (0.14–0.26)**	/	78.2 %	0.91 (0.70–1.17)	0.83 (0.62–1.10)
CD4<200	94.0 %	**0.09 (0.06–0.13)**	**0.09 (0.06–0.14)**	/	74.6 %	1.10 (0.87–1.41)	1.03 (0.78–1.37)
Time since HIV diagnosis							
<1 year	54.2 %	1	1	1	49.2 %	1	1
1 – <5 years	78.3 %	**0.33 (0.28–0.39)**	**0.32 (0.27–0.39)**	**0.26 (0.21–0.33)**	81.5 %	**0.22 (0.18–0.27)**	**0.22 (0.17–0.27)**
5 – <10 years	89.3 %	**0.14 (0.12–0.17)**	**0.13 (0.11–0.16)**	**0.12 (0.07–0.19)**	88.4 %	**0.13 (0.10–0.16)**	**0.12 (0.09–0.15)**
≥10 years	94.5 %	**0.07 (0.06–0.08)**	**0.06 (0.05–0.07)**	/	85.9 %	**0.16 (0.13–0.20)**	**0.14 (0.11–0.18)**

*p* < 0.05, statistically significant variables presented in boldface

^a^Adjusted for sex, age at diagnosis, nationality, way of transmission

^b^Additional adjustment for CD4 value at first visit (only includes patients diagnosed between 2006 and 2011, *n* = 2437)

Multivariate analyses show that younger age remained associated with lower ART uptake and less VL suppression. HIV testing requested for clinical reasons and lower CD4 count at first contact remained associated with higher ART uptake and Sub-Saharan African nationality with less VL suppression. Those with longer time from diagnosis had higher ART uptake and more frequent VL suppression. Multivariate analyses on ART uptake were adjusted for CD4 count at first medical visit. As time since ART initiation was not available, it could not be taken into account in the multivariate analyses on VL suppression.

### Proportions of diagnosed patients along the continuum of HIV care in Belgium

We estimated that 11,478 patients were retained in HIV care in 2011 by applying the proportion of retention in HIV care of 92.2 % observed in 2010 to the 12,449 HIV+ patients with at least one laboratory record in 2011.

The proportion of late entry in care of 4.7 % observed in the 2007 data was applied to persons diagnosed in the years following 2007. After subtraction of those already entered in care, we found that 221 patients were expected to enter in care after 2011 (see detailed computation in Additional file 1).

Re-entry in care among patients not retained in care for 1 year after 2007 was estimated at 39.5 %. By applying this proportion of re-entry to the patients not retained in care in the following years and then subtracting those already re-entered in care, we estimated that 940 persons were expected to re-enter in care after 2011 (see detailed computation in Additional file 1).

After combining these results, the population of diagnosed HIV patients living in Belgium at the end of 2011 (*n* = 12,639) was distributed as follows: 11,478 patients (90.8 %) were estimated to be retained in HIV care, 221 (1.8 %) were not linked to HIV care and 940 (7.4 %) were not retained in HIV care.

The estimated proportions of 84.6 % of patients in care on ART of whom 83.4 % of patients with viral suppression were applied to the population in HIV care in 2011 (*n* = 12,449). We estimated 83.3 % (*n* = 10,532) of the diagnosed HIV population living in Belgium to be on ART and 69.5 % (*n* = 8784) to have suppressed VL.

**Fig. 3:**
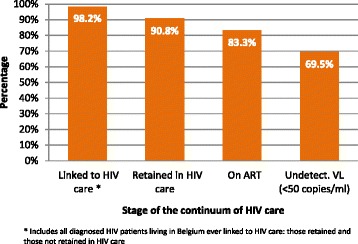
Estimated percentage of diagnosed HIV individuals living in Belgium by stage of the continuum of HIV care, 2011

The continuum of HIV care of the diagnosed HIV patients living in Belgium is illustrated in Fig. 3.

## Discussion

The continuum of HIV care in Belgium presents low attrition rates over all stages of care with 69 % of all HIV diagnosed individuals in Belgium having an undetectable viral load.

HIV care leans on well-organized specialized structures, the ARLs and the ARCs, that offer access to comprehensive care including free psycho-social support, counselling and fully reimbursed antiretroviral therapy. In addition, these specialized structures have developed collaborations with prevention and testing associations that contribute to a more efficient linkage to HIV care.

Vulnerable populations such as non-Belgians and IDU present lower entry and retention in HIV care but, once retention in care is ensured, their access to ART are similar to other HIV-infected individuals. Yet, poorer virological outcome was observed among SSA migrants. Similar findings for lower entry and retention of these populations are reported in other European studies [13, 16, 17], and lower ART uptake or viral suppression among migrants was observed in Spain [18], UK [19] and France [20]. IDU represent only 2.5 % of the HIV-infected patients in care in Belgium with a sustained low HIV transmission reported over the last years [5] due to accessible harm reduction programs. The limited number of IDUs enables more intensive follow-up and counselling to prevent comorbidities and onward HIV transmission by unsafe injecting practices.

Additionally, more frequent delayed diagnoses observed in these two populations (migrants and IDUs) worsen the poorer outcomes observed along the continuum of care [5, 21]. For migrants whose infection was acquired in their country of origin, the diagnosis delay may precede the arrival in Belgium.

Migrants’ lower entry and retention in HIV care may be due to emigration after HIV diagnosis or barriers to access care for those remaining in Belgium. Attrition is similar between Europeans and non-Europeans, but consequences of emigration out of Belgium in terms of access and continuum of care greatly differ. While West-Europeans have reliable access to care in their country of origin, this may not be true for East-European and non-European migrants. In fact, reported ART coverage among those in need of ART in numerous non-European or East-European countries was below 50 % in 2012 [22] and potentially even lower for stigmatized populations like MSM and IDU. This study does not allow differentiation between documented and undocumented migrants although it is mainly undocumented migrants that face disproportionate barriers to medical care [10]. The HIV epidemic in Europe is influenced by its evolution in other countries through travel and migration [23]. The continuum of care for this mobile population could be improved by developing cross-border and national tools to facilitate adequate and individual-tailored care of the migrant population diagnosed with HIV in Belgium.

The results also underline a gap in linkage to care among patients diagnosed for pre-operative reasons due to longer delay between initial diagnosis and entry in care. Timely communication of HIV diagnosis to the patient in specific settings like pre-operative testing would be facilitated by training of caregivers on sexual health counselling and HIV screening. These interventions are part of the recommendations of the Belgian National HIV plan that has been launched in 2013 [24].

Factors impacting ART uptake and VL suppression were logical indicators of the clinico-immunological situation of the patient: duration since HIV diagnosis, older age with associated co-morbidity, HIV diagnosis for clinical reason and low CD4 count. None of the socio-demographic factors studied impacted ART uptake and VL suppression, except the SSA nationality that was associated with lower VL suppression, although the difference with Belgians was small. These results suggest that ARV treatment needs are equally covered for all patients having access to regular HIV care. Suboptimal ART adherence potentially related to socio-economic factors [18] might explain the slightly lower VL suppression among SSA. Other reasons for non VL suppression might be ARV resistance or recent ART initiation at the time of VL measurement but this information was not collected.

Patients on ART have a higher retention in care as also observed in another cohort [17].

The attrition observed along the continuum of HIV care should not be interpreted globally but according to the stage at which it occurs. Indeed, its consequences, both in terms of individual prognosis and potential for HIV transmission are different in early and late stages of the continuum of care. In the early stages of the continuum, HIV-infected individuals who are not diagnosed, not linked or not retained in care have a higher risk of delay in ART initiation, complications and co-morbidities and have no access to regular psycho-social support and counselling. Hence, attrition in the early stages is associated with a higher likelihood of unfavourable clinical evolution and onward transmission [25]. In the late stages of the continuum, patients retained in regular HIV care in Belgium have access to counselling services and timely ART initiation based on their viro-immunological status and their presumed need for ART to prevent transmission. Those untreated or presenting detectable VL among this carefully monitored population are considered as lost in the late stages of the continuum of care according to the cascade analysis definition. Yet, the likelihood of them causing onward transmission or having an unfavourable clinical evolution is lower than among the population lost in the early stages of the continuum [26].

In other European cascades of HIV care, reported proportions of VL suppression among those diagnosed with HIV ranged from 70 % (<500 copies/mL) in Sweden and Denmark in 2010 [13], 72 % (<50 copies/mL) in UK in 2011 [12] and 64 % (<50 copies/mL) in France in 2010 [14]. Given the various definitions used for the study population and for each stage of the continuum of care, international comparison is difficult. Nevertheless Belgian results are close to previously reported European proportions while differing strongly from the recently reported proportion of 35 % of those diagnosed achieving viral suppression (<200 copies/mL) in the United States in 2011 [11].

The UNAIDS has recently published global targets of 90 % of PLHIV knowing their status, 90 % of those receiving ART and 90 % of those having suppressed VL by 2020 [27]. The results presented here show that Belgium is not yet fulfilling these targets. With the recent release of the results of the START study supporting added benefit of offering treatment to everyone with HIV [28] however, we may expect an increase in the proportion of patients on ART in the coming years. This might also increase the retention in HIV care among recently diagnosed patients, who will initiate ART immediately, as ART uptake was associated with higher retention in HIV care. Efforts should then concentrate on the diagnosis of persons unaware of their HIV infection in order to initiate their treatment earlier.

Our understanding of the continuum of HIV care is limited by the lack of precise national estimates of PLHIV unaware of their status. In Europe, 30 % of PLHIV were estimated to be unaware of their infection, with large variations between countries [23]. In Belgium, a local survey limited in sample size and geographical coverage, reported a proportion of undiagnosed HIV-infected MSM of 14 % in 2010 [8]. In the national STI sentinel network, the proportion of patients co-infected with HIV and STI ignoring their HIV status was 14 % in 2011 [29]. Among migrants newly diagnosed in 2013, 93 % of those with known date of arrival (70 %) came to Belgium in the 2 year period preceding the diagnosis in Belgium [30], implying a limited period of residence in Belgium as undiagnosed. Based on these various sources of information, the proportion of undiagnosed PLHIV residing in Belgium is probably not higher than 20 %. This approximate estimate is similar to the proportions of 24 and 19 % found in UK and France respectively [14, 31, 32]. As those infected and remaining undiagnosed for long end up being diagnosed late, the similar proportion of late diagnoses in Belgium as compared to these two countries [8], suggests that the pool of undiagnosed PLHIV in Belgium is not larger than in surrounding countries. When including this estimate in the continuum of care, the proportion of individuals with suppressed VL among all PLHIV residing in Belgium is estimated at 56 %. The proportion of 20 % of PLHIV unaware of their HIV infection could represent the weakest stage of the Belgian HIV care continuum. Given the low attrition observed along the subsequent steps of the continuum of HIV care, identifying the undiagnosed PLHIV could be the intervention with potentially the highest impact on transmission and patient prognosis in Belgium, this coincides with observations made in other countries such as UK, Canada and the US [25, 33]. A project aiming at estimating and characterizing undiagnosed HIV-infected populations by mathematical modelling of routine HIV surveillance data will be conducted in the coming years. These results will be used to target HIV testing to groups that require it the most. Among those undiagnosed, recently HIV-infected individuals appear to be disproportionately involved in onward HIV transmission, likely due to increased viral concentrations, higher viral fitness and more risky behaviour around time of HIV acquisition [2, 34]. In Belgium, the proportion of recently infected individuals among those diagnosed was estimated at 37.5 % in 2012 [35], higher than in UK and France where it ranged from 22 to 30 % [14, 31, 32]. HIV testing strategies, tailored to identify those recently infected as well as those who have remained undiagnosed for long, should be maintained and expanded. Alternative decentralized testing strategies should be further developed along with interventions to reduce missed opportunities for earlier HIV diagnosis in medical settings.

There are some limitations in this study. The results do not inform on the outcomes of the patients after they have left the country. In addition, HIV-infected citizens of surrounding countries are also difficult to capture in the continuum of care as they might be diagnosed and followed in their own country whilst actively contributing to the local epidemic.

We used 2007 data to estimate the proportions of late entry and re-entry into HIV care as time of follow-up for these patients was at least 4 years. We assumed these proportions did not change over the next years and applied them to estimate the number of patients with late entry and with re-entry during the following years. However as linkage to care within 1 year improved slightly after 2007, the proportion of patients expected to enter late in HIV care in the following years might be lower. Hence the population of persons diagnosed and not linked to HIV care living in Belgium might be slightly overestimated based on the proportions estimated in 2007.

This analysis was strongly dependent of a good match between HIV diagnosis data and laboratory data through identifiers built on patients’ date of birth and initials. Errors on these identifiers are not excluded but they are likely to represent a very low proportion of the entire dataset thanks to the thorough annual monitoring.

The analyses of the early stages of the continuum of care included all the individuals diagnosed with HIV and in care in Belgium. Only the analyses on ART coverage and VL suppression were restricted to the patients retained in care in the ARCs. The ARC population differed significantly from those outside of the ARCs for some characteristics such as gender (women: 36 % vs 42 %), nationality (Sub-Saharan Africans: 36 % vs 43 %; Belgians: 50 % vs 45 %), mode of transmission (MSM: 40 % vs 30 %; heterosexuals: 54 % vs 56 %) and median age (34 years vs 32 years). Given the small proportion of patients retained in care outside the ARCs (20 %) and age being the single characteristics associated with ART uptake and VL suppression, the estimates obtained from the ARC population could be used as estimates for the whole study population.

Information on age and sex was missing in less than 0.5 % of the cases, data on mode of transmission and nationality were missing for approximately 30 % of cases, and up to 60 % for those who never entered in care. The high proportion of missing information among the latter is foreseeable given their medical contact limited to the diagnosis consultation. Such cases included a majority of patients who emigrated after diagnosis as any other patient remaining in Belgium would end up entering in care, thus higher completeness of such data would probably reinforce the strong association observed between no entry and non-Belgian nationality.

## Conclusions

The continuum of HIV care in Belgium is well ensured although some barriers to access care for migrants, possibly linked with their legal status, are observed. Migrants who emigrated after diagnosis or those who are in care in neighbouring countries were not captured by this analysis, although these are major actors in the dynamic of the epidemic. Strategies to improve identification of the undiagnosed PLHIV is a priority. Together with the improvement of the HIV care continuum of migrants, these strategies would contribute to move closer to the ambitious “90-90-90” global targets of the UNAIDS [27].

## Additional file

Additional file 1:
**Computation for estimates of populations in HIV care.** (DOCX 14 kb)

## Abbreviations

ARC: AIDS Reference Centre
ARL: AIDS Reference Laboratory
ART: Antiretroviral therapy
IDU: Injecting drug use
MSM: Men who have sex with men
PLHIV: People living with HIV
VL: Viral load
WIV-ISP: Scientific Institute of Public Health
