# Supplementary material for: Good continuum of HIV care in Belgium despite weaknesses in retention and linkage to care among migrants
Source: BMC Infect Dis. 2015 Nov 3;15:496. doi: 10.1186/s12879-015-1230-3 (PMC4631021; doi:10.1186/s12879-015-1230-3)
Supplement: Additional file 1: — Computation for estimates of populations in HIV care. (DOCX 14 kb) [file 12879_2015_1230_MOESM1_ESM.docx]

Estimates of the populations diagnosed with HIV living in Belgium:

The population living in Belgium diagnosed with HIV and not linked to HIV care at end of 2011 and the population living in Belgium and not retained in HIV care at end of 2011 were estimated as follows:

**Estimation of the population diagnosed with HIV and not linked to HIV care living in Belgium:**

A proportion of late entry in care of 4.7% was estimated using the data of 2007. This proportion was applied to the persons diagnosed in the years following 2007 and after subtraction of those already entered in care by end of 2011, we found numbers of patients expected to enter in care after 2011 of 24, 41, 44 and 52 for 2008, 2009, 2010 and 2011 respectively.

Using the proportion of 89% of linkage to HIV care found in 2010, we calculated the number of persons diagnosed in 2011 and expected to enter in HIV care within one year of diagnosis, then we subtracted those already entered in HIV care, and we obtained and additional 63 persons.

This sums up to a total of 221 persons living in Belgium with diagnosed HIV but not linked to HIV care.

**Estimation of the population not retained in care living in Belgium:**

Among those not retained in care after 2007, 39.5% did re-enter in care in the following years. We applied this proportion to the patients not retained in care in the years following 2007 as shown in the table.

Also, we obtained an estimate of 971 patients in care in 2011 and not retained, by using the proportion of 7.8% of no retention found in 2010. On these 971 patients estimated to be not retained in 2011, we estimated that 39.5% would re-enter after 2011, this represents 383 patients.

These numbers sum up to a total of 940 HIV+ persons expected to re-enter HIV care after 2011.

Table: Computation for the estimates of the number of patients expected to re-enter in HIV care after 2011.

| Year of care | Nr of patient not retained | Nr of patients expected to re-enter in HIV care | Nr of patients re-entered in care by 2011 | Nr of patients expected to re-enter in HIV care after 2011 |
| --- | --- | --- | --- | --- |
| 2007 | 752 | 297 | 297 | 297-297=0 |
| 2008 | 823 | 39.5%*823= 325 | 305 | 325-305=20 |
| 2009 | 986 | 39.5%*986=389 | 237 | 389-237=152 |
| 2010 | 976 | 39.5%*976=385 | 0 | 385 |
| 2011 | 7.8% * 12449 = 971 | 39.5%*971=383 | 0 | 383 |
| Total |  |  |  | **940** |
